# Flexible nanopillar-based electrochemical sensors for genetic detection of foodborne pathogens

**DOI:** 10.1186/s40580-018-0147-0

**Published:** 2018-06-06

**Authors:** Yoo Min Park, Sun Young Lim, Soon Woo Jeong, Younseong Song, Nam Ho Bae, Seok Bok Hong, Bong Gill Choi, Seok Jae Lee, Kyoung G. Lee

**Affiliations:** 1 Nano-bio Application Team, National NanoFab Center (NNFC), Daejeon, 34141 Republic of Korea; 2 Division of Advanced Materials Science and Engineering, Hanbat National University, Daejeon, 34158 Republic of Korea; 3 Department of Chemical Engineering, Kangwon National University, Samcheok, 25913 Republic of Korea

**Keywords:** Lithography, Nanopillar arrays, Nanopillar electrode, Foodborne illnesses, Genetic analysis

## Abstract

Flexible and highly ordered nanopillar arrayed electrodes have brought great interest for many electrochemical applications, especially to the biosensors, because of its unique mechanical and topological properties. Herein, we report an advanced method to fabricate highly ordered nanopillar electrodes produced by soft-/photo-lithography and metal evaporation. The highly ordered nanopillar array exhibited the superior electrochemical and mechanical properties in regard with the wide space to response with electrolytes, enabling the sensitive analysis. As-prepared gold and silver electrodes on nanopillar arrays exhibit great and stable electrochemical performance to detect the amplified gene from foodborne pathogen of *Escherichia coli* O157:H7. Additionally, lightweight, flexible, and USB-connectable nanopillar-based electrochemical sensor platform improves the connectivity, portability, and sensitivity. Moreover, we successfully confirm the performance of genetic analysis using real food, specially designed intercalator, and amplified gene from foodborne pathogens with high reproducibility (6% standard deviation) and sensitivity (10 × 1.0^1^ CFU) within 25 s based on the square wave voltammetry principle. This study confirmed excellent mechanical and chemical characteristics of nanopillar electrodes have a great and considerable electrochemical activity to apply as genetic biosensor platform in the fields of point-of-care testing (POCT).

## Introduction

As continuous worldwide growth of food industry, rapid detection of foodborne pathogens has been brought great interests in public health surveillance in a human’s daily life [1–3]. Trade of contaminated food between communities and/or countries continuously increase health risk and microbial pathogens in food are of major concerns to all governments [4, 5]. Although conventional detection method of culture and colony counting method and immunology-based method are proposed, the great amount of population suffers from foodborne diseases every years [6–10]. Moreover, rapid and early detection of pathogenic microbial is still considered as the most important but difficult tasks because of requirement of collaboration of multiple disciplinary including food microbiology, chemistry, clinical medicine, and laboratory medicine and demands of complex handling processes, experts, complicated detection method, and expensive equipment [11–14]. Therefore, the next generation of detection tool kits with nanotechnology incorporated lightweight, portable, and flexible/wearable biosensor platforms have been brought great attentions for realization of on-site analysis of specific target bioanalytes including microorganisms, protein, toxins, and nucleic acids [15–18].

Recently, flexible sensors have received a great deal of attention as a point of care testing (POCT) platforms for the continuous monitoring of foodborne illness. POCT technologies offers reduce operating time, simplicity, easy-to-use, portability, non-expertise, relatively cheap, and rapid decision making [19–22]. Of the various materials used to date, polymer is one of the most widely used flexible and bendable substrate in human’s daily life because of its low permeability, low electrical conductivity, excellent chemical stability, strong mechanical stability, and functionality [23–26]. These unique characteristics make polymer a compatible platform for the construction of flexible electrochemical biosensors. To construct of highly flexible and bendable electrochemical biosensors, polymer nanopillar arrays have been proposed and demonstrated its excellent performance [27–29]. In particular, the conductive nanopillar arrays enable to realize high surface area, high aspect ratio, enhancement of mass transfer, easy control of surface functionalization offer great potential as electrode materials in electrochemical sensing applications [30–32]. Using the combination of polymerase chain reaction (PCR) and electrochemical sensing. Among them, electrochemical sensors offers simple, portable, and effective detection of genetic level of foodborne pathogens is urgent and important to prevent outbreak of foodborne diseases [33].

Herein, inspiring from the previous findings, we developed a polyaniline nanopillar array-based flexible/bendable electrochemical sensors for genetic analysis of foodborne pathogens. Combination of polymeric blend and photo-/soft-lithography allowed us to prepare flexible/bendable nanopillar array substrate. The sensing electrode was fabricated via patterning of gold layers on nanopillar array substrate and used as a working and counter electrodes while silver was patterned as a reference electrode. Produced nanopillar arrayed electrode were applied in an electrochemical sensor and showed excellent electrochemical performances. The as-prepared electrochemical sensors demonstrated excellent genetic detection with benefits of simple fabrication, scalability, high sensitivity, good reproducibility and specificity using realistic foodborne pathogen models. Additionally, it showed considerable potential as genetic biosensor platform for POCT.

## Experimental

### Materials and apparatus

UV polymerizable NOA63 (Norland Optic Adhesives), PU (MINS-311RM, Minuta Tech.), and PET film (MITSUBISHI, Japan) were purchased and used without further purification. GoTaq^®^DNA polymerase (M3005) was from Promega. *E. coli* O157:H7 (ATCC 43894) was obtained from ATCC. QuickExtract™ DNA extraction solution 1.0 (QE09050) was purchased from Epicentre. 2′-(4-Hydroxyphenyl)-5-(4-methyl-1-piperazinyl)-2,5′-bi-1H-benzimidazole trihydrochloride hydrate, bisBenzimide (Hoechst 33258), and 2′-(4-hydroxyphenyl)-5-(4-methyl-1-piperazinyl)-2,5′-bi-1H-benzimidazole trihydrochloride hydrate, bisBenzimide (HEPES) were obtained from Sigma-Aldrich. The thermal cycler (C1000Touch™) device was obtained from Bio-Rad. UV visualizer (LIAS Slite 140) was obtained from Avegene Life Sciences. The DNA extraction and purification solution (QIAamp DNA mini kit, 51304) was purchased from QIAGEN. The electrochemical evaluation was carried out with the 630B (CH Instruments). The electron beam evaporator (EBS400) was purchased from EVATEC.

### Fabrication of nanopillar electrodes

Nanopillar arrays were initially fabricated on the Si wafers by using of photo/soft-lithography followed by oxygen penetration and dry etching, producing the cylindrical shapes with diameters of 500 nm and heights of 1500 nm. Then, the composition of polyurethane acrylate (PU) and NOA63 (NO) as 7:3 (v/v) was selected in the study based on the previous report [27, 28]. To fabricate the flexible substrate, the PUNO was detached from the wall of the Si mold. The formation of thin titanium and gold layers by vacuum sputtered on the surface of nanopillar arrays with assistance of stencil mask. As prepared gold electrodes applied as working and counter electrode. Then, silver electrode was further printed on the nanopillar using silver paste and it was used as a reference electrode.

### Structure and electroactivity characterization of nanopillar electrode

The structure of nanopillar arrays on the electrode was evaluated by scanning electron microscopy (SEM). The electrochemical behavior of nanopillar was determined by cyclic voltammetry (CV) scanning the voltage from − 0.1 to 1.6 V at 50 mV/s in the 50 mM H_2_SO_4_. The NPE stability was investigated by scanning the scan rate from 10 to 500 mV/s and 30 time CV cycling at the 10 and 50 mV/s using the 5 mM ferrocyanide.

### *E. coli* O157:H7 preparation

The *E. coli* O157:H7 was cultured in the Luria–Bertani (LB) broth including the sodium chloride, yeast extract, and tryptone in 100 mL distilled water at 37 °C for 18 h. By using the colony counting method, the cells number suspended in the broth was estimated. The suspended *E. coli* O157:H7 were spiked into a broth or milk sample concentration from 1.0 × 10^1^ to 1.0 × 10^5^ per 100 µL broth or 1 mL milk. The each prepared *E. coli* O157:H7 samples were stored at 4 °C until use. The gDNA from each cells was extracted and purified for evaluation.

### Polymerase chain reaction (PCR) condition for *E. coli* O157:H7

The genomic sequences of eaeA gene of *E. coli* O157:H7 was from GenBank. The forward primer was 5′-GACCCGGCACAAGCATAAGC-3′, and the reverse primer was 5′-CCACCTGCAGCAACAAGAGG-3′. The total amplified gene sizes were 384 bp. The extracted and purified gene of *E. coli* O157:H7 was reacted with a PCR mixture containing the dNTP (dATP, dGTP, dCTP, and dTTP), MgSO_4_, forward and reverse primer, and polymerase. The following PCR thermocycling procedures was employed: 95 °C for 300 s (pre-denaturation) and 30 cycles of 95 °C for 30 s (denaturation of double-stranded DNA template), 60 °C for 30 s (annealing of each primer to the single-stranded DNA template), 72 °C for 30 s (synthesizing the DNA strand complementary to the template strand by polymerase), and 72 °C for 300 s (final elongation).

### Nanopillar-based electrochemical detection of *E. coli* O157:H7

The amplified gene of *E. coli* O157:H7 was mixed with the Hoechst electrolyte, which specifically intercalates with double-stranded DNA. The electrochemical determination was implemented by analyzing the current in a final Hoechst concentration at 33.3 μM solution. The mixture of Hoechst with amplified gene was assessed to the NPE by square wave voltammetry (SWV) method. The potential scanning from 0 to 0.5 V was performed under 0.025 V amplitude and 25 Hz frequency at 50 mV/s within 25 s.

## Result and discussion

### Manipulation and structure confirmation of flexible nanopillar electrode (NPE)

**Fig. 1:**
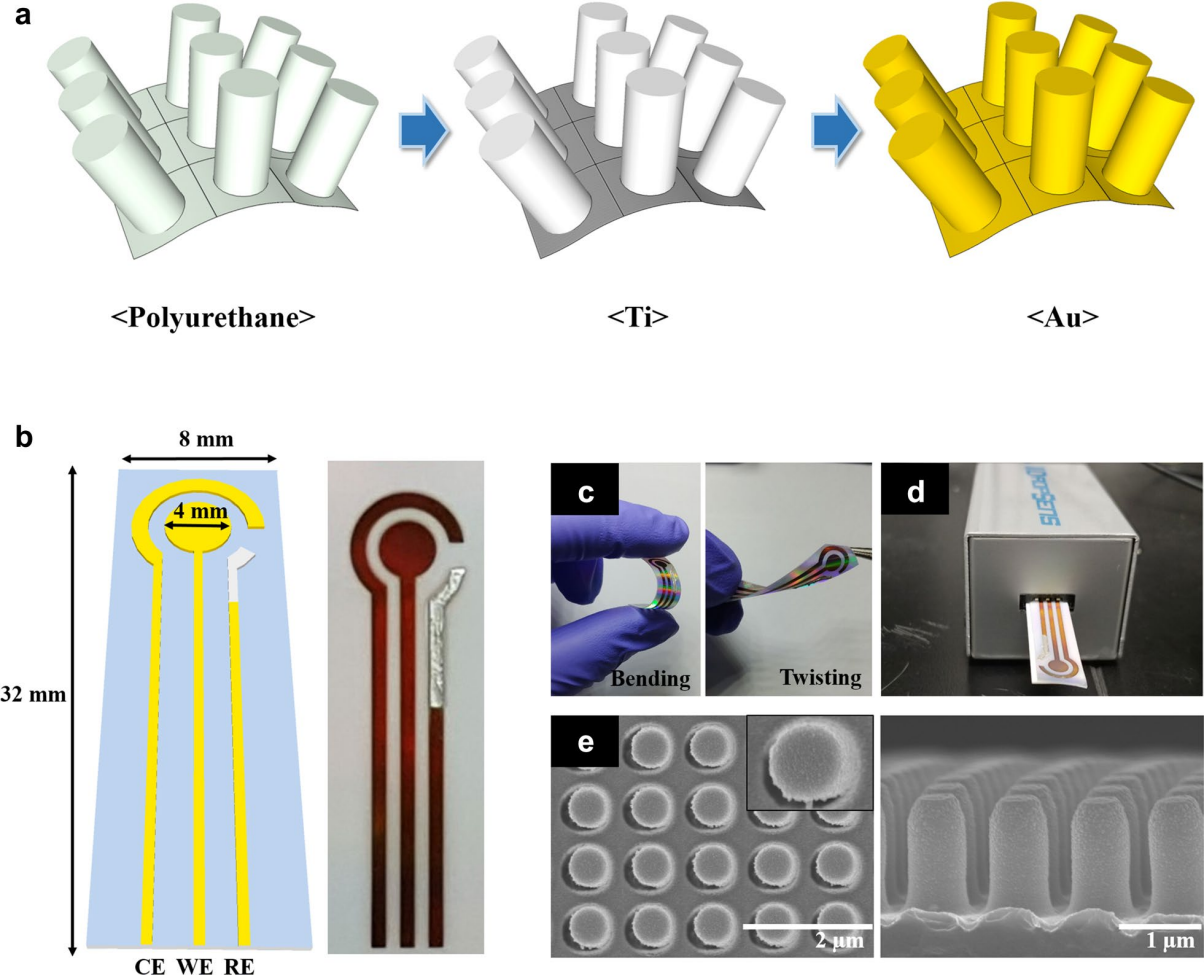
**a** Schematic illustration of NPE fabrication processes. **b** Scheme and photographic image of NPE. Photographic images of **c** bending and twisting status of NPE and **d** confirmation of USB connection. **e** SEM images of top and side view of NPE

The flexible nanopillar electrodes (NPE) were typically manipulated in two steps as follows: (1) the nanopillar array-constructed polyurethane (PU) was prepared by employing the conventional photo- and soft-lithography principle. (2) The titanium (Ti) and gold (Au) layer was then sequentially deposited onto the PU substrate using the electron beam (e-beam) evaporation with assistance of metal stencil mask. The overall processes is schematically illustrated as shown in Fig. 1a. The USB connectable three electrode allowed for the easy connection and analysis of electrochemical signals. As shown in Fig. 1b, the counter electrode (CE) and working electrodes (WE) consist of gold while the reference electrode (RE) is silver paste. The detailed schematic design of NPE demonstrates and its width and length were 8 and 32 mm with the diameter of 4 mm of WE, respectively (Fig. 1b, left). The thin layer of Au and silver electrodes were successfully formed on the transparent film as shown in Fig. 1b (right). The thin film NPE exhibited excellent flexibility and NPE was highly stably to the external deformation such as bending and twisting (Fig. 1c). Additionally, there were almost no visible damages against to the external stress and the results indicated that the diverse structure and conformation is possible in comparison with rigid type electrode based on the silicon or glass. Considering the portability and accessibility for on-site point-of-care testing (POCT), the NPE was designed as the conventional USB and the electrochemical analysis was further confirmed the electrochemical performance using portable connector (Fig. 1d).

In this study, the 500 nm width and 1500 nm height nanopillar was produced with the center-to-center distance of 1000 nm. To verify the uniformity of the nanopillar structure, scanning electron microscopy (SEM) was performed to investigate the morphology of sensor surface as shown in Fig. 1e. The SEM image revealed that the nanopillar was equally arranged in the length and breadth, and the same height of nanopillar arrays were also uniformly produced [27]. Additionally, the flat gold deposition on the surface was observed. Based on the obtained result, the designed NPE was accurately manipulated with high reproducibility, and the electroactivity of NPE was further carried out using electrochemical experiment.

### Characterization of nanopillar electrode

**Fig. 2:**
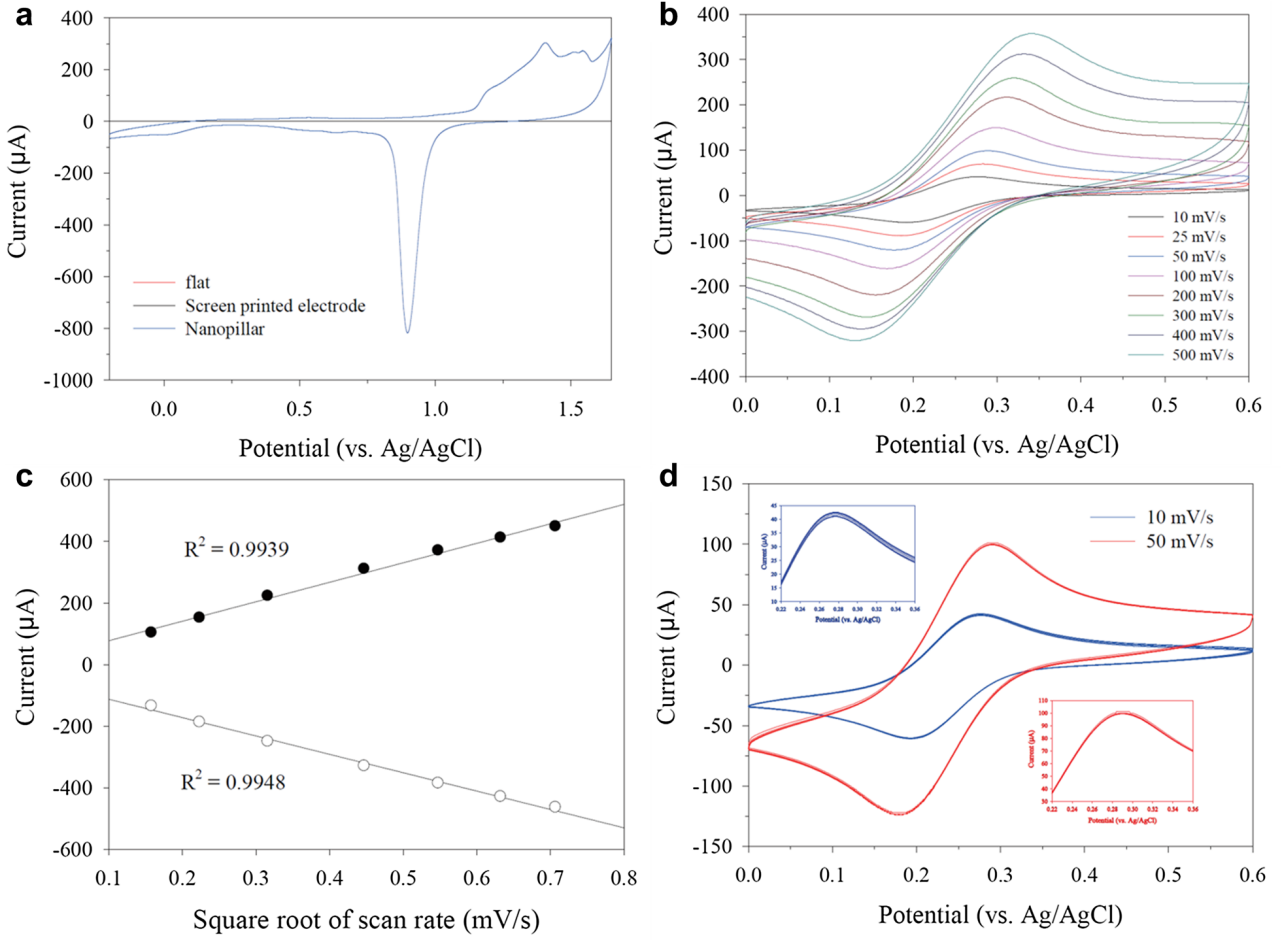
**a** The electrochemical behavior of nanopillar electrode in comparison with flat electrode and screen printed electrode using the H_2_SO_4_. **b** Cyclic voltammogram of NPE in the scan rate range from 10 to 50 mV/s. **c** Redox peak current from the (**b**). Reproducibility test of NPE at the 10 and 50 mV/s. The 5, 10, 15, 20, 25, and 30 cycle is presented

The electrochemical characterization of fabricated NPE was intensively investigated. The 50 mM H_2_SO_4_-based test was firstly conducted, and the flat gold electrode and conventional screen printed electrode (SPE) were compared. As shown in Fig. 2a, the cyclic voltammetry (CV) curve of NPE obviously exhibited oxidation peak and sharp reduction peak (vs Ag/AgCl). However, no prominent redox peaks detected at flat electrode and SPE. The result was mainly attributed to the NPE transferred relatively higher current response because of the high efficient and rapid electron transfer characteristics of nanotopography in comparison with the flat electrode and SPE. To verify the electrochemical behavior of NPE, the ferrocyanide-based CV was further carried out in various scan rate. As a result, the redox peak current was sequentially changed on increasing the scan rate from 10 to 500 mV/s (Fig. 2b) without irregular responses in whole applied range. Meanwhile, the each redox peak current signals linearly increased in accordance with the increase of scan rate. Based on the obtained redox peak signal, the oxidation peak and reduction peak of the calculated linearity of R^2^ values were 0.9939 and 0.9948, respectively (Fig. 2c). Based on the result, the gold-deposited NPE demonstrated the remarkable electrochemical behavior of the redox reaction on the nanopillar surface. For the investigation of the reproducibility, the CV was repeatedly performed under scan rate of 10 and 50 mV/s at least 30 times. Figure 2d presented the voltammogram for 5, 10, 15, 20, 25, and 30 cycle for each scan rate. The homogeneous electrochemical signals were registered in the both condition. The magnified oxidation peak current area showed in the inlet on Fig. 2d to precisely investigate the signal variation. As shown in the result graph, the signal level was approximately similar in whole applied condition. Based on the findings, the obtained reliable result for electrochemical characterization reveals the high stability of NPE.

### Electrochemical evaluation of *E. coli* O157:H7

**Fig. 3:**
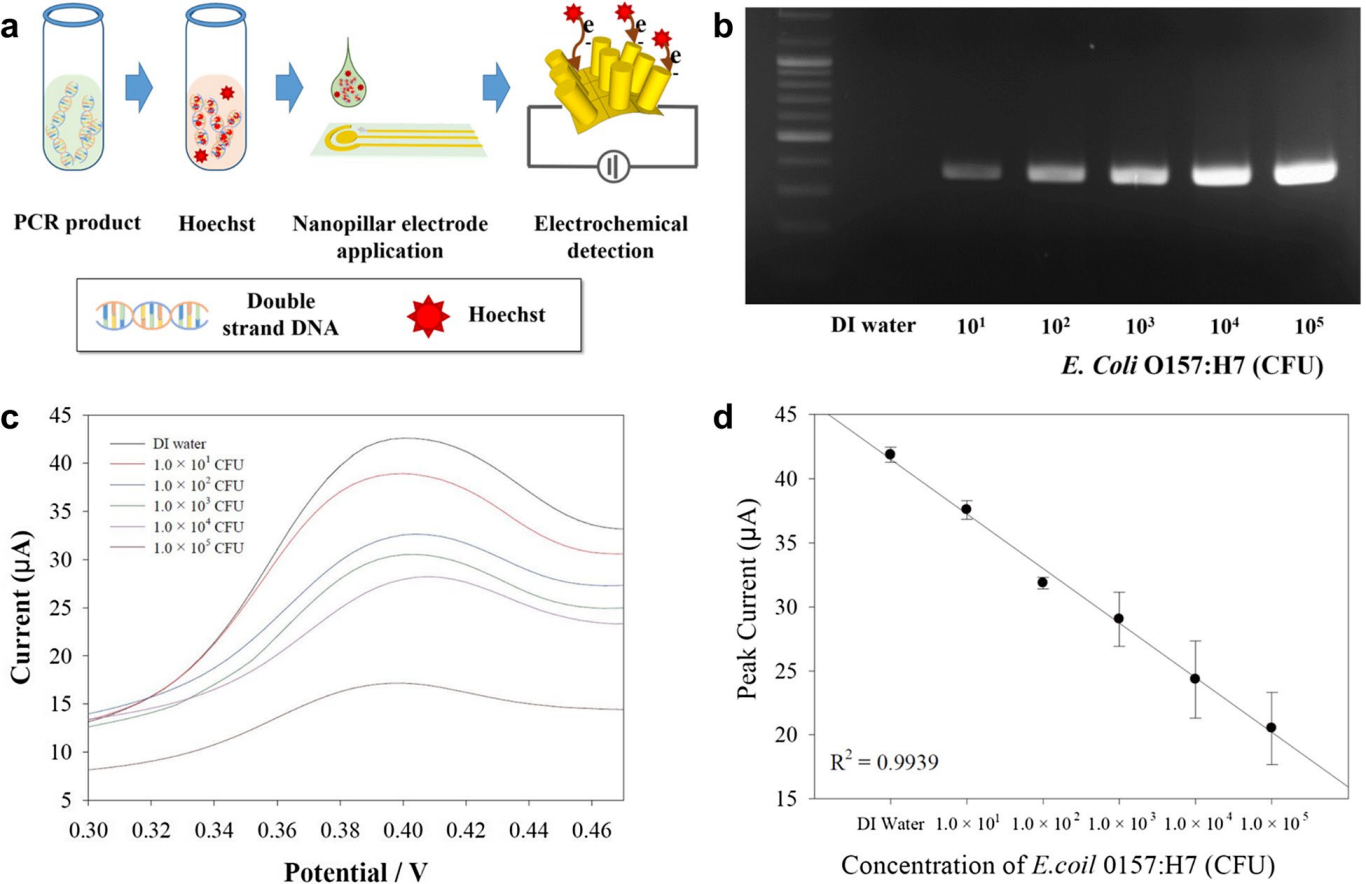
**a** Illustration of NPE-based electrochemical evaluation of *E. coli* O157:H7. **b** Optical image of gel electrophoresis from the amplified gene of *E. coli* O157:H7 by conventional PCR. **c** The voltammogram from each applied test. **d** Calibration curve of electrochemical analysis of *E. coli* O157:H7 using the NPE. The same tests were repeated at least three times, and the error bar indicated the signal variation

In regard with the food poisoning caused by a small amount of bacteria in the food, the various concentrations of *E. coli* O157:H7 from 1.0 × 10^1^ to 1.0 × 10^5^ CFU were prepared and electrochemically evaluated using as-prepared NPE (Fig. 3). The whole and detailed experiment processes exhibited in Fig. 3a. Briefly, the extracted genes from different amount of *E. coli* O157:H7 were amplified by conventional PCR then, the PCR product were mixed with the Hoechst for electrochemical analysis. The Hoechst is specifically designed to intercalate with double strand DNA, while the Hoechst could not selectively react with specific DNA species. Thus, to selectively analyze the target pathogen, the particularly designed primer should be employed for the gene of target pathogen regarding the international standard base pair sequence. The whole mixture solution of PCR product from different amount of *E. coli* O157:H7 and Hoechst was applied on to the surface of as-prepared NPE and carefully analyzed the individual electrochemical signal changes (Fig. 3a). Prior to the quantification and investigation of electrochemical signal of *E. coli* O157:H7, the visualization of conventional gel electrophoresis confirmed the successfulness and relative quantification of PCR product as shown in Fig. 3b. The intensity of the PCR bands also gradually increased as corresponding to the concentration of *E. coli* O157:H7 while no optical signal observed from DI water test.

In order to demonstrate the electrochemical performance of NPE, the mixture of amplified pathogen gene and Hoechst were prepared and applied to investigate the sensing capability. As the pristine Hoechst inducing the electrochemical signals, the obtained signal was reversely proportional to the target gene concentration. The registered electrochemical signal from *E. coli* O157:H7 test was measured and presented in Fig. 3c, the voltammogram from each test was gradually increased in accordance of *E. coli* O157:H7 concentration. The calibration curve was presented based on the peak current as shown in Fig. 3d. In the test result, a good linear relationship as corresponding to the target concentration represented that the introduced Hoechst-based signaling principle was properly performed with NPE. In the calibration curve, the signals from 1.0 × 1.0^1^ to 1.0 × 10^5^ CFU of *E. coli* O157:H7 demonstrated a linear response. The obtained electrochemical test results were highly correlated with optical signal from Fig. 3b. This result strongly supported the reliability of the developed NPE-based biosensing system. Additionally, the developed sensing system could be able to serve as the new analytical system on behalf of the conventional gel electrophoresis method. The conventional optical detection method required about 30 min while the developed sensing system could rapidly analyze the target gene within 25 s, effectively reducing the 1/70-fold detection time. The calibration curve exhibited the saturation at the high concentration of *E. coli* O157:H7, while the signals were clearly distinguished in the linear detection range, indicating the high sensitivity of nanopillar electrode to the small amount of target gene [24]. Based on the obtained test result, the limit of detection for *E. coli* O157:H7 evaluation was 1.0 × 10^1^ CFU which is the clear evidence of high sensitivity of the nanopillar-based sensing system. To verify the reproducibility of the NPE to detect the *E. coli* O157:H7, the intra- and inter-assay was implemented at least three times under same experimental condition such as pH, temperature, and reaction time (Fig. 3d). The calculated coefficient of variation (COV) was about 10% for the whole *E. coli* O157:H7 test. The correlation coefficient in the linear detection range from 1.0 × 1.0^1^ to 1.0 × 10^5^ CFU test also confirmed, and the calculated R^2^ value was 0.99 because of the high reproducibility and reliability of NPE. Based on the findings, the test result demonstrated that the developed NPE-based sensing system could be a stable and reliable platform to detect foodborne pathogen of *E. coli* O157:H7.

### Real sample-based *E. coli* O157:H7 analysis

**Fig. 4:**
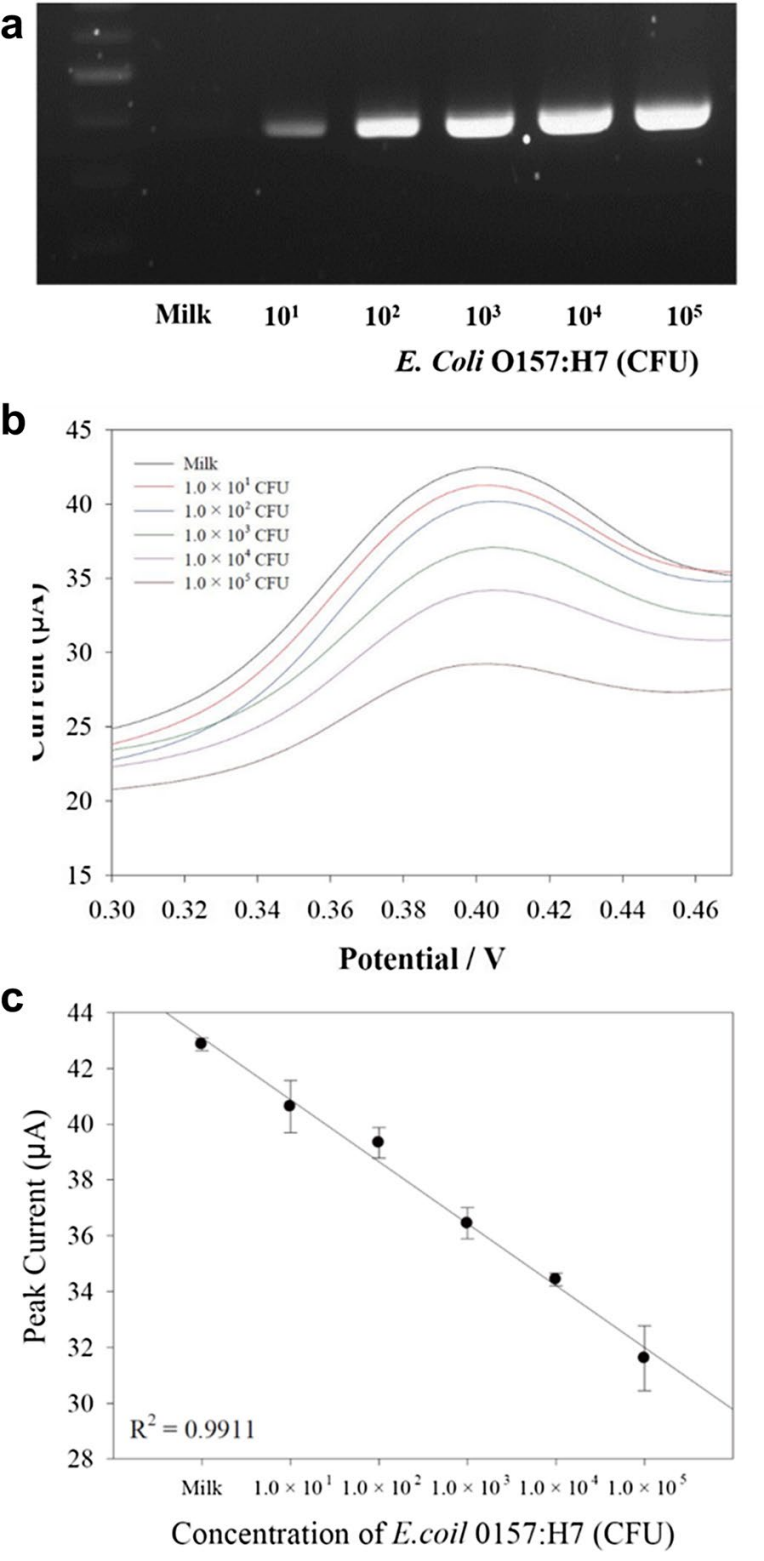
**a** The gel electrophoresis image of milk-spiked *E. coli* O157:H7, **b** the NPE-based electrochemical responses to the milk-spiked *E. coli* O157:H7 concentration from 1.0 × 10^1^ to 1.0 × 10^5^ CFU and **c** the calibration curve. The tests were repeatedly conducted under same condition. The signal variation was presented in the error bar

To determine the practical utility of the NPE, the milk-based *E. coli* O157:H7 evaluation was implemented. Briefly, the *E. coli* O157:H7 concentration from 1.0 × 10^1^ to × 10^5^ CFU added to the 1 mL of milk. The mixture solution of pathogen and milk were pre-treated and amplified by PCR under same condition with previous principle [33]. In Fig. 4a, the electrophoresis gel images showed the intensity changes as responding to the *E. coli* O157:H7 concentration and it confirmed the proper amplification of target gene in the milk. After amplification, The Hoechst was added to the PCR product of pathogen-spiked milk and evaluate the performance of NPE as responded to the amount of pathogens in the milk. The presented electrochemical signal of voltammogram in Fig. 4b were correlated to the each applied *E. coli* O157:H7 concentration. Based on the obtained electrochemical signals, the calibration curve was registered in Fig. 4c. The result graph demonstrate the linear calibration curve in whole detection range. To verify the correlation coefficient, the calculated R^2^ value was 0.99 that is a clear evidence of the high sensitivity of NPE [27, 33]. Based on the obtained result, the 1.0 × 10^1^ CFU is the LOD in the milk-based *E. coli* O157:H7 determination. The calculated R^2^ value was 0.99 and this indicated the high reproducibility and reliability of NPE to the milk-based evaluation. Despite of high linearity, the signal level in the whole detection range is higher than that with previous result. The probable reason is that the intercalation of Hoechst to the amplified gene could be interfered with ingredient in the milk such as albumin, casein, and proteins [34]. Consequently, the relatively high concentrations of pristine Hoechst generate the electrochemical signals on the NPE surface.

The signal variation to the intra- and inter-assay was further carried out. The prepared *E. coli* O157:H7-spiked milk was repeatedly estimated under same condition including the pH, temperature, and reaction time. The error bar in Fig. 4b indicate the signal variation to the each *E. coli* O157:H7 evaluation. The calculated COV in whole obtained result is 6% and it is the reasonable reproducibility of NPE to the real food-based *E. coli* O157:H7 analysis. In regard with the requirements of sensitive and rapid analysis of pathogens causative of foodborne illnesses, the developed NPE successfully accomplished the feasibility performance for molecular diagnostic system to use in POCT.

## Conclusions

We demonstrated a reliable process for fabrication of flexible and highly arrayed NPE-based sensing platforms by using photo-/soft-lithography and a subsequent electrode patterning process. The NPE showed a great mechanical stability and electrochemical properties. Additionally, USB connectable design allows us to realize easy accessibility and in situ analyze the existence of foodborne pathogens via detection of electrochemical signals. We also investigated the biosensing performance using real food with pathogenic *E. coli* O157:H7 based on intercalation of Hoechst and amplified gene. The NPE-base electochemical sensors exhibited high sensitivity and specific detection of foodborne pathogen. As-developed flexible NPE sensing platform could provide portability, low-cost, reliability, and disposable sensing platform to realize on-site POCT biosensors.

## References

[1] Kang D-K, Ali MM, Zhang K, Huang SS, Peterson E, Digman MA, Gratton E, Zhao W (2014). Nat. Commun..

[2] Rissin D, Kan CW, Campbell TG, Howes SC, Fourniner DR, Song L, Piech T, Patel PP, Chang L, Rivnak AJ, Ferrel EP, Randall JD, Provuncher GK, Walt DR, Duffy DC (2010). Nat. Biotechnol..

[3] Sharma H, Mutharasan R (2013). Sens. Actuators B Chem..

[4] Baer AA, Miller MJ, Dilger AC (2013). Compr. Rev. Food Sci. Food Saf..

[5] Cho I-H, Ku S (2017). Int. J. Mol. Sci..

[6] Allen MJ, Edberg SC, Reasoner DJ (2004). Int. J. Food Microbiol..

[7] Van DE, Leven M, Pattyn S, Van Damme L, Laga M (2001). J. Clin. Microbiol..

[8] Taylor AD, Yu Q, Chem S, Homola J, Jiang S (2005). Sens. Actuators B.

[9] Arora P, Sindhu A, Dilbaghi N, Chaudhury A (2011). Biosens. Bioelectron..

[10] Perumal V, Hashim U (2014). J. Appl. Biomed..

[11] Kim YT, Park KJ, Kim S, Kim SA, Lee SJ, Kim DH, Lee TJ, Lee KG (2018). Talanta.

[12] Abdalhai MH, Fernandes AM, Bashari M, Ji J, He Q, Sun X, Agric J (2014). Food Chem..

[13] Mehta J, Vinayak P, Tuteja SK, Chhabra VA, Bhardwaj N, Paul AL, Kim K-H, Deep A (2016). Biosen. Bioelectron..

[14] Zhan W-W, Xu J-J, Chem H-Y (2014). Chem. Rev..

[15] Su L, Jia W, Hou C, Lei Y (2011). Biosens. Bioelectron..

[16] Mehrotra P (2016). J. Oral Biol. Craniofac. Res..

[17] Turner APF (2013). Chem. Soc. Rev..

[18] Ping J, Vishnubhotla R, Vrudhula A, Johnson ATC (2016). ACS Nano.

[19] John AS, Price CP (2014). Clin. Biochem. Rev..

[20] Kim I, Moon J-S, Oh J-W (2016). Nano Converg..

[21] Yamada K, Suzuki K, Citterio D (2017). ACS Sens..

[22] Ren X, Yan J, Wu D, Wei Q, Wan Y (2017). ACS Sens..

[23] Yang M, Kim DS, Yoon JH, Hong SB, Jeong SW, Yoo DE, Lee TJ, Lee SJ, Lee KG, Choi BG (2016). Analyst.

[24] Shin C, Shin W, Hong HG (2007). Electrochim. Acta.

[25] Wolfrum B, Mourzina Y, Mayer D, Schwabb D, Offenhäusser A (2006). Small.

[26] Chen J-K, Zhou G-Y, Chang C-J, Cheng C-C (2014). Sens. Actuators B Chem..

[27] Lee KG, Choi BG, Kim BI, Shyu T, Oh MS, Im SG, Chang S-J, Lee TJ, Kotov NA, Lee SJ (2014). Adv. Mater..

[28] Yang MH, Jeong SW, Chang SJ, Kim KH, Jang M, Kim CH, Bae NH, Sim GS, Kang T, Lee SJ, Choi BG, Lee KG, Appl ACS (2016). Mater. Interfaces.

[29] Windmiller JR, Wang J (2013). Electoanalysis.

[30] Zheng Y, Jiao Y, Jaroniec M, Jin Y, Qiao SZ (2012). Small.

[31] Zhu C, Yang G, Li H, Du D, Lin Y (2015). Anal. Chem..

[32] Reta N, Saint CP, Michelmore A, Prieto-Simon B, Voelcker NH, Appl ACS (2018). Mater. Interfaces.

[33] Cinti S, Volpe G, Piermarini S, Delibato E, Palleschi G (2017). Sensors.

[34] Steijns JM (2001). Int. J. Dairy Technol..

